# Does the coronoid fracture in terrible triad injury always need to be fixed?

**DOI:** 10.1186/s12893-024-02394-3

**Published:** 2024-04-25

**Authors:** Yeong-Seub Ahn, Seong-Hwan Woo, Sungmin Kim, Jun-Hyuk Lim, Tae-Hoon An, Myung-Sun Kim

**Affiliations:** 1 Department of Orthopaedic Surgery, Chonnam National University Hospital, Chonnam National University College of Medicine, 42 Jebong-ro, Dong-gu, Gwangju, 61469 South Korea; 2 Department of Orthopaedic Surgery, Good Morning General Hospital, Pyeongtaek, Republic of Korea

**Keywords:** Coronoid fracture, Elbow, Fixation, Height loss ratio, Intraoperative elbow stability test, Outcomes

## Abstract

**Background:**

The ideal treatment of terrble triad injuries and whether fixation of coronoid process fractures is needed or not are still debated. Therefore, we aimed to investigate if terrible triad injuries necessitate coronoid fracture fixation and evaluate if non-fixation treatments have similar efficacies and outcomes as fixation-treatments in cases of terrible triad injuries.

**Methods:**

From August 2011 to July 2020, 23 patients with acute terrible triad injuries without involvement of the anteromedial facet of the coronoid process were included to evaluate the postoperative clinical and radiological outcomes (minimum follow-up of 20 months). According to the preoperative height loss evaluation of the coronoid process and an intraoperative elbow stability test, seven patients underwent coronoid fracture fixation, and the other eight patients were treated conservatively. The elbow range of motion (ROM), Mayo Elbow Performance Score (MEPS), and modified Broberg-Morrey score were evaluated at the last follow-up. In addition, plain radiographs were reviewed to evaluate joint congruency, fracture union, heterotopic ossification, and the development of arthritic changes.

**Results:**

At the last follow-up, the mean arcs of flexion-extension and supination-pronation values were 118.2° and 146.8° in the fixation group and 122.5° and 151.3° in the non-fixation group, respectively. The mean MEPSs were 96.4 in the fixation group (excellent, nine cases; good, tow cases) and 96.7 in the non-fixation group (excellent, ten cases; good, two cases). The mean modified Broberg-Morrey scores were 94.0 in the fixation group (excellent, sevev cases; good, four cases) and 94.0 in the non-fixation group (excellent, ten cases; good, tow cases). No statistically significant differences in clinical scores and ROM were identified between the two groups. However, the non-fixation group showed a significantly lower height loss of the coronoid process than the fixation group (36.3% versus 54.5%).

**Conclusions:**

There were no significant differences in clinical outcomes between the fixation and non-fixation groups in terrible triad injuries.

## Background

The term “terrible triad of the elbow” was first used to describe injuries combining elbow posterior dislocation with disruption of the lateral ulnar collateral ligament (LUCL) in association with fractures of the radial head and coronoid process [1]. Historically, these complex injuries have been difficult to treat, with variable outcomes [1–3]. Most of these injuries are surgically treated to restore early elbow stability and recover joint mobility [1, 4]. Over time, various studies on surgical management have been conducted. Most operative management advocates fixing the radial head and coronoid process fracture as well as repairing the elbow ligament to achieve stable elbow joint motion [3–10]. Likewise, most authors prefer to attempt fixation of the fractured coronoid process [7–9, 11–14].

The ideal treatment and whether fixation of coronoid process fractures is required or not are still debated. With respect to these aspects, some studies were conducted to demonstrate the stability of terrible triad injuries of the elbow without fixation of Regan-Morrey type I or II coronoid process fractures [15, 16]. One cadaveric study suggested that Regan-Morrey type I and II fractures were stable when the radial head was not resected [15]. However, this cadaveric study had a limitation in that it was a biomechanical study, not a clinical study on surgically treated patients with terrible triad injuries. In another study on Regan-Morrey type I and II coronoid fractures [16], Papatheodorou et al. suggested that terrible triad injuries can be successfully managed without coronoid fracture fixation; their study was performed on patients who did not undergo coronoid fracture fixation, and the results were compared to those of other studies in which patients underwent coronoid fixation.

Based on these findings, we postulated that elbow stability could be achieved without coronoid fracture fixation when the lateral structures, including the radial head and lateral ligament complex, are restored in Regan-Morrey type II fractures of terrible triad injuries. Therefore, we retrospectively evaluated patients with terrible triad injury who underwent surgical treatment at a center and performed clinical and radiological comparisons between patients with and without coronoid fracture fixation.

## Methods

### Ethics statements

The local institutional review board approved the patient record review and all the collected data. Because of the retrospective nature of the study, the requirement for formal consent was waived.

### Study design and population

A retrospective review of the patients who sustained acute terrible triad injuries and were surgically treated from August 2011 to July 2020, was conducted. The patients who underwent surgical treatment and had at least one year of outpatient follow-up period recorded were included to evaluate the clinical and radiological outcomes. Among 27 patients, 2 patients were excluded from this study because they had a concomitant distal humerus or forearm fracture, and another 2 patients were excluded because the minimum follow-up period after surgery was not one year. Twenty three patients who met the inclusion criteria with a minimum follow-up of 20 months (mean, 57 months; range, 20–104 months) were retrospectively reviewed. Among the 23 patients, 11 patients underwent surgical coronoid fracture fixation (fixation group), and the other 12 patients received conservative treatment without fixation (non-fixation group).

### Evaluation of height loss

**Fig. 1:**
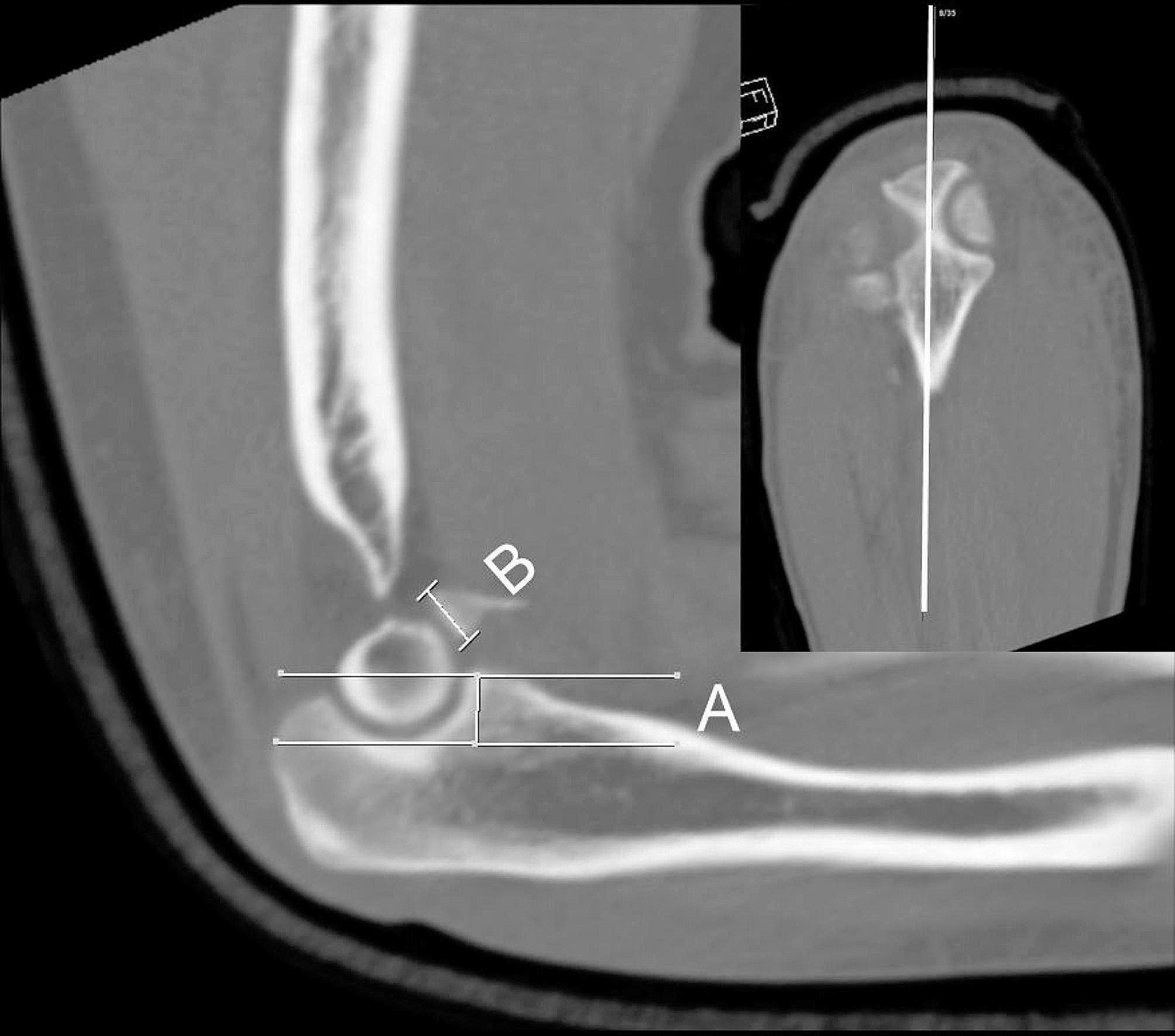
Preoperative evaluation of height loss of the coronoid process using computed tomography (CT). Height loss of the coronoid process is evaluated using a preoperative CT study. After the midline portion at the coronal plane is identified, it is used as a reference to confirm the mid-sagittal plane of the coronoid process. In the mid-sagittal plane, the height loss of the coronoid process is evaluated by measuring the ratio of the height of the fractured fragment (**b**) to the total expected coronoid process height (**a + b**)

The height loss of the coronoid fracture was assessed using preoperative three-dimensional computed tomography (CT) scans. The mid-portion at the coronal plane of the proximal ulna was identified and used as a reference to confirm the mid-sagittal plane of the coronoid process. In the mid-sagittal plane, the height loss of the coronoid process was measured, as shown in Fig. 1. The height loss ratio was evaluated as the height of the fractured fragment to the total expected coronoid process height.

### Treatment

**Fig. 2:**
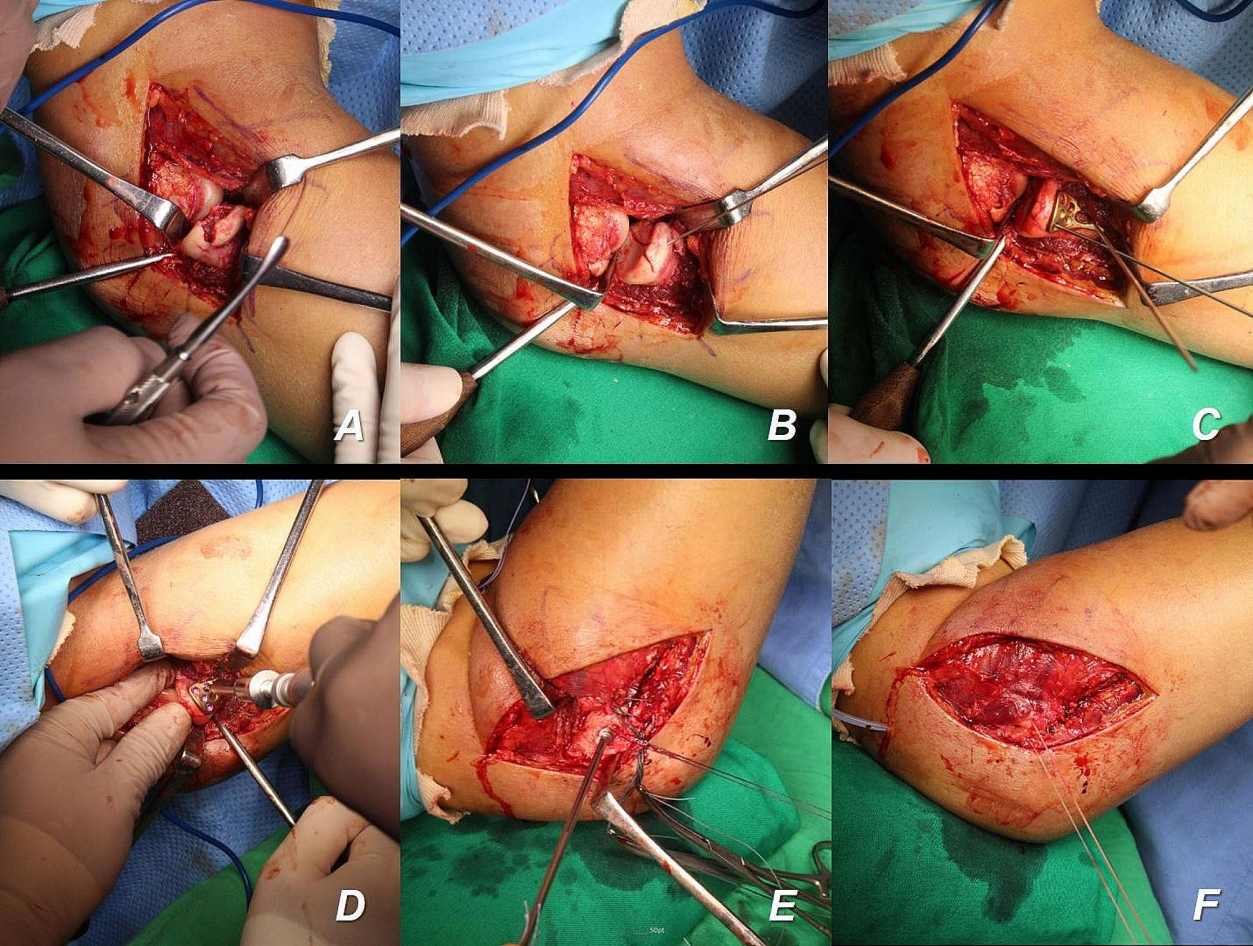
Intraoperative clinical photographs of a patient treated without coronoid fixation. Using the lateral approach, the fracture of the radial head fracture is identified first (**a**). Temporary Kirschner wire fixation is performed after radial head reduction (**b**). Then, plate fixation is performed to maintain reduction of the fracture site (**c, d**). After stable fixation of the radial head is achieved, the lateral ulnar collateral ligament complexes are repaired using the suture anchor (**e, f**)

**Fig. 3:**
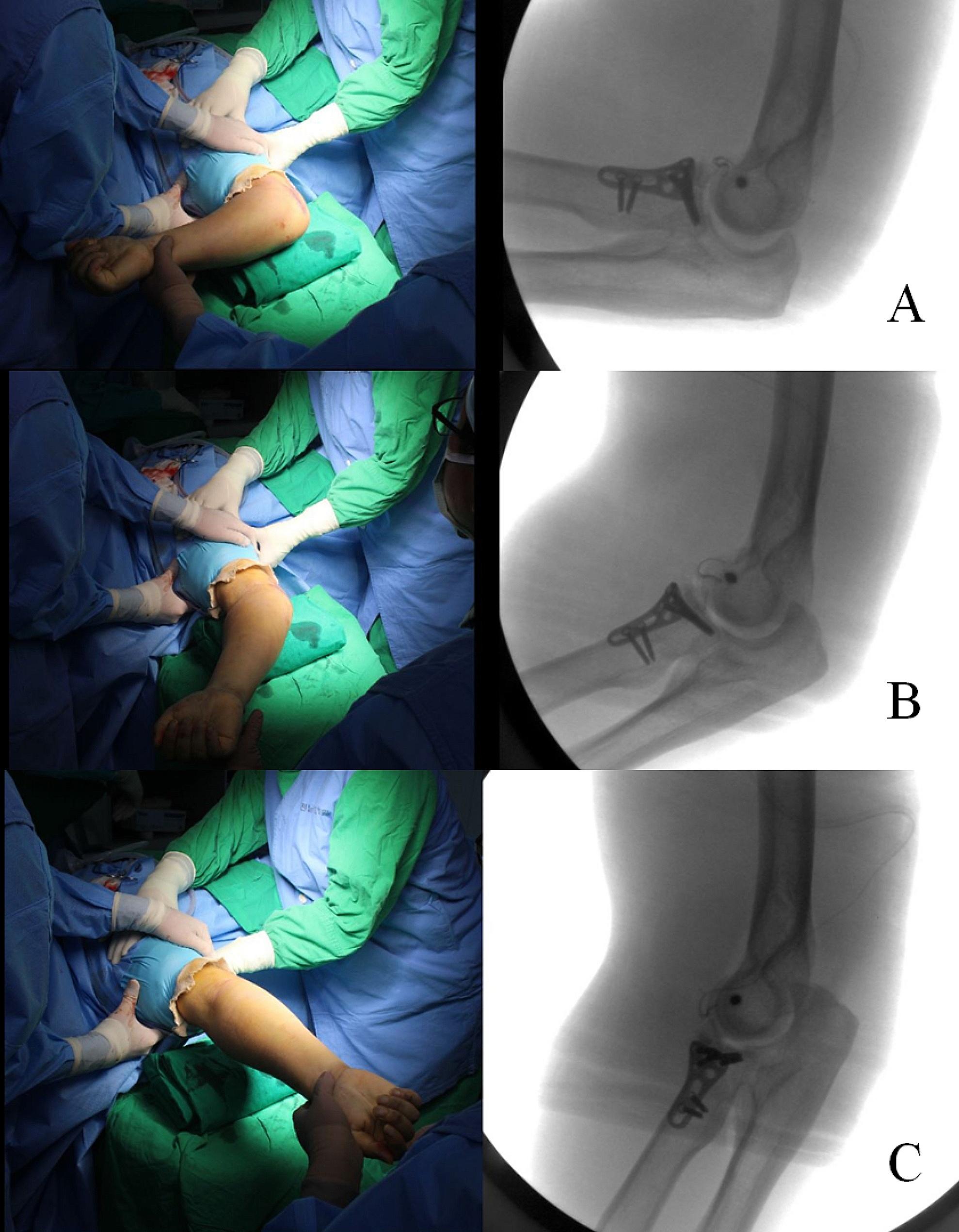
Intraoperative stability test method using fluoroscopy. After the radial head fracture and lateral ulnar collateral ligament injury are repaired, the stability test using fluoroscopy is performed during elbow motion at 90° to 20° of flexion-extension. As shown, this patient maintained the concentric reduction while the elbow was flexed and extended (**a–c**)

All 23 patients underwent radial head replacement or osteosynthesis for radial head fracture and LUCL repair. However, fixation of the coronoid process fracture differed depending on the severity of the fracture. The decision for coronoid fracture fixation was made based on the preoperative height loss evaluation of the coronoid process using CT and the intraoperative stability test. On preoperative CT evaluation, if the height loss of the coronoid process exceeded 50% of the total height, internal fixation was performed. However, if the height loss was less than 50% of the total height (Fig. 1), an intraoperative stability test was preferentially planned to determine whether to fix the coronoid fracture or to perform additional management for medial ligament injury after radial head and LUCL repair (Fig. 2a–f). The result of the intraoperative stability test using fluoroscopy was assessed during elbow motion from 90° to 20° of flexion-extension (Fig. 3a–c). If concentric reduction was maintained while the elbow was flexed and extended, we considered that elbow stability could be achieved without coronoid fracture fixation or additional management of the medial ligament complex.

**Fig. 4:**
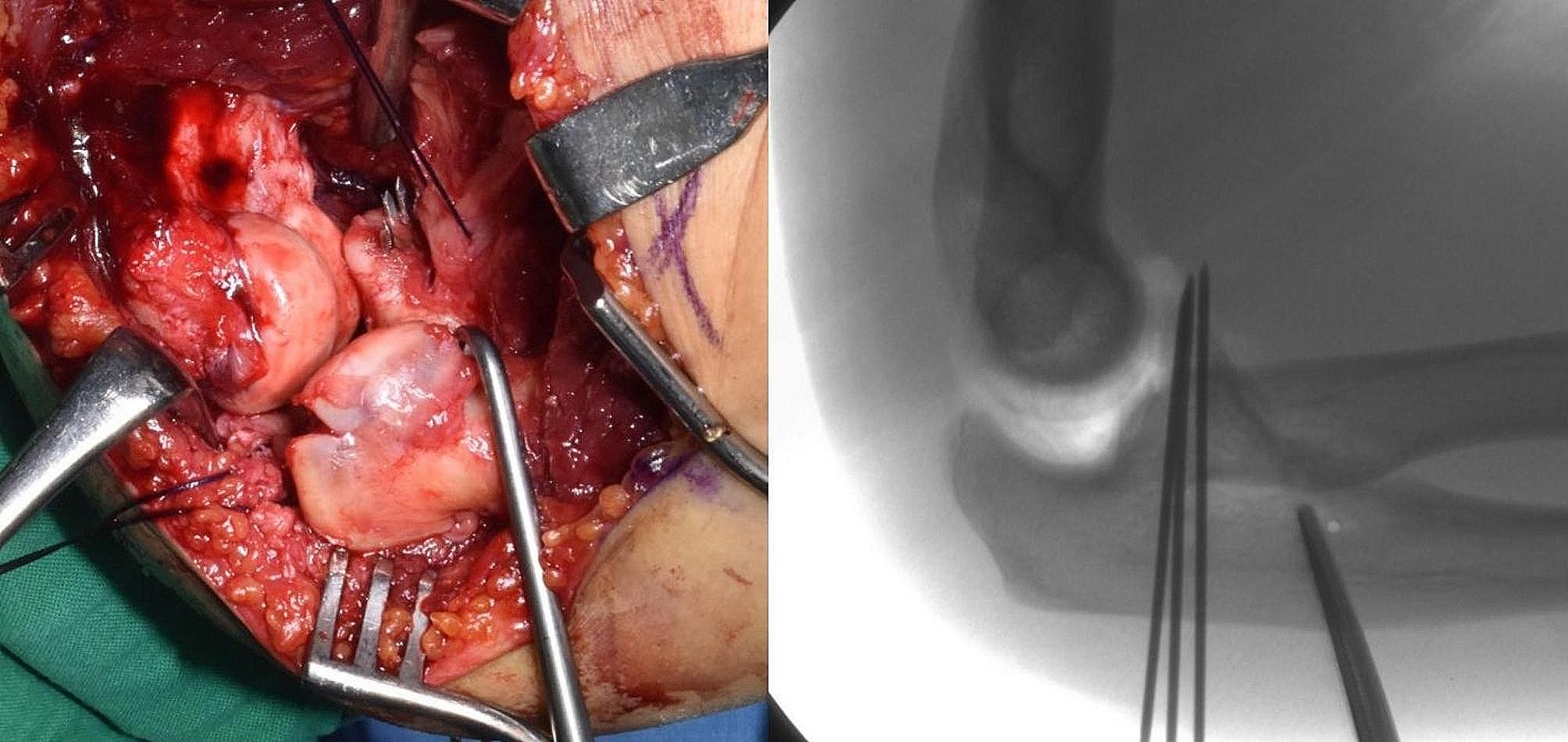
Fixation method of the coronoid process through the lateral approach. In cases in which the height loss of the coronoid process is expected to exceed 50%, reduction is performed through the lateral window formed by the radial head fracture site with percutaneous posterior Kirschner wire fixation

In the fixation group, nine of eleven patients were confirmed to have medial collateral ligament (MCL) injury by magnetic resonance imaging (MRI) scans. Among those, two patients were identified to have concomitant common flexor origin injury. six of seven patients with an intact common flexor origin were treated with the lateral approach. For coronoid fractures, reduction was performed through the lateral window formed by the radial head fracture site, and percutaneous posterior Kirschner wire fixation was performed (Fig. 4). In the other patient with a relatively large coronoid deficiency, radial head and coronoid fixation were performed through a common posterior approach. After coronoid fixation was completed, osteosynthesis and arthroplasty were performed for the radial head fracture, respectively, and the LUCL was finally repaired. Subsequently, elbow stability was confirmed through an intraoperative stability test using fluoroscopy, and no additional surgery for MCL injury was performed. In addition, in one of two other patients who were confirmed to have concomitant common flexor origin rupture, lateral structures (the radial head fracture and LUCL injury), including the coronoid fracture, were fixed only through the lateral approach. However, concentric reduction was not maintained in the intraoperative elbow stability test during extension. Therefore, common flexor origin and MCL ruptures were repaired through the additional medial approach. In the other patient, plate fixation for the coronoid fracture and common flexor origin and MCL repair were performed together through the medial approach.

In the non-fixation group, ten of twelve patients were confirmed to have an MCL injury by MRI scans. Among them, one patient had a concomitant common flexor origin rupture and a radial head fracture and LUCL injury, which were first addressed through the lateral approach alone. However, the elbow stability test showed an unstable elbow joint, so the additional medial approach was performed to repair the common flexor origin and MCL rupture. For the other patients without common flexor origin ruptures, operative management was performed using only the lateral approach to restore the lateral structures, and stability was maintained without surgical repair of the MCL.

### Rehabilitation and follow-up

All patients received the same rehabilitation program. After 2 weeks of immobilization at 90° in a long-arm splint with slight forearm pronation, a hinged brace was applied to initiate protective range of motion (ROM) exercise.

### Outcome assessment

At follow-up, clinical and radiological evaluations were performed for each patient. Elbow ROM was evaluated in two planes: sagittal motion (extension and flexion) and coronal rotation (pronation and supination) were measured to one decimal range using the electronic goniometer. Clinical outcomes were evaluated using the Mayo-Elbow Performance Score (MEPS) and modified Broberg-Morrey score. Elbow stability was assessed by clinical examination after at least six weeks of postoperative follow-up. At each follow-up, plain radiographs were reviewed to evaluate joint congruency, fracture union, heterotopic ossification, and the development of arthritic changes.

### Endpoints

Do terrible triad injuries necessitate coronoid fracture fixation?

Do non-fixation treatments have similar efficacies and outcomes as fixation-treatments in cases of terrible triad injuries?

### Statistical analysis

Statistical analysis was performed using IBM SPSS Statistics for Windows version 21.0 (released 2012; IBM Corp., Armonk, NY, USA). The Kruskal-Wallis test was used to compare clinical data between the fixation and non-fixation groups. Statistical significance was ascribed to *P* < 0.05.

## Results

The mean age of 23 patients (20 males, 3 female) was 54 years. The mechanism of injury was a fall from height in most patients except one (bicycle traffic accident). There was no significant difference in age at the time of surgery, number of dominant hand injuries, duration from injury to surgical treatment, and postoperative follow-up duration between the groups (Table 1). The rate of height loss of the coronoid process was higher in the fixation group than in the non-fixation group (Table 2).

**Table 1:** Comparison of demographic data between the fixation and non-fixation groups

	Fixation group	Non-fixation group	*P* value
No. of patients (sex)	(9 M/2 F)	(11 M/1 F)	55
Age at surgery (years)	0.1 (30.0–73.0)	0.3 (34.0–83.0)	803
Number of dominant hand injury	(64%)	(58%)	
Time duration at trauma to surgical treatment (days)	(1.0–14.0)	0.3 (1.0–14.0)	912
Postoperative follow-up duration (months)	0.3 (20.4–79.9)	0.1 (24.9–104.9)	101

**Table 2:** Comparison of coronoid deficiency ratio measured by height loss of fractured coronoid between the fixation and non-fixation groups

	Fixation group	Non-fixation group	*P* value
Height loss (%)	2.5 (50.1–60.3)	6.3 (20.7–47.7)	0.001

With respect to the evaluation of sagittal elbow motion, in the fixation group, the mean elbow flexion at the last follow-up was 125.1° (range, 110.0°–140.0°), whereas the mean flexion contracture was 5.5° (range, 0.0°–20.0°), with a mean arc of sagittal motion (flexion/extension) of 118.2° (range, 90.0°–140.0°). In the non-fixation group, the mean elbow flexion at the last follow-up was 127.5° (range, 110.0°–140.0°), whereas the mean flexion contracture was 5.0° (range, 0.0°–20.0°), with a mean arc of sagittal motion (flexion/extension) of 122.5° (range, 105.0°–140.0°). As for the evaluation of elbow coronal rotation, in the fixation group, the mean supination was 79.1° (range, 50.0°–90.0°), whereas the mean pronation was 67.7° (range, 45.0°–90.0°), with a mean arc of coronal rotation (supination/pronation) of 146.8° (range, 95.0°–180.0°). In the non-fixation group, the mean supination was 80.8° (range, 70.0°–90.0°), whereas the mean pronation was 70.4° (range, 45.0°–90.0°), with a mean arc of coronal motion (supination/pronation) of 151.3° (range, 130.0°–180.0°). The overall arc of motion in both groups was included in a functional range, and we observed comparable results without significant difference between the groups (Table 3).

**Table 3:** Comparison of postoperative range of motion at latest follow-up period between the fixation group and non-fixation group

	Fixation group	Non-fixation group	*P* value
Sagittal plane			
Flexion	5.1 (110.0–140.0)	27.5 (110.0–140.0)	510
Extension	5 (0.0–20.0)	0.0 (0.0–20.0)	973
Arc of Flex. / Ext.	18.2 (90.0–140.0)	2.5 (105.0–140.0)	480
Coronal plane			
Supination	0.1 (50.0–90.0)	0.8 (70.0–90.0)	0.999
Pronation	0.7 (45.0–90.0)	0.4 (45.0–90.0)	683
Arc of Sup. / Pro.	46.8 (95.0–180.0)	1.3 (130-0–180.0)	664

In terms of clinical outcomes, the mean MEPSs were 96.4 (range, 85.0–100.0) and 96.7 (range, 85.0–100.0) in the fixation and non-fixation groups, respectively. According to the MEPS, the fixation group had nine excellent results and two good results, and similarly, the non-fixation group had ten excellent results and two good results. In addition, the modified Broberg-Morrey score was comparable between the groups with mean scores of 94.0 (range, 83.0–100.0) and 94.0 (range, 85.0–100.0), respectively. Although the number of patients with excellent results was higher in the non-fixation group than in the fixation group, no significant difference was observed (Table 4).

**Table 4:** Comparison of postoperative clinical score evaluated by MEPS and modified Broberg-Morrey score during the latest follow-up period between the fixation and non-fixation groups

	Fixation group	Non-fixation group	*P* value
MEPS	96.4 (85.0–100.0)	96.7 (85.0–100.0)	4
Excellent	9	10	99
Good	2	2	
Modified Broberg-Morrey score	94.0 (83.0–100.0)	94.0 (85.0–100.0)	999
Excellent	7	10	371
Good	4	2	

MEPS, Mayo Elbow Performance Score

On radiographic review, all patients who underwent osteosynthesis for radial head fracture showed bony union, and none of the patients treated with radial head replacement showed implant failure. Although one patient underwent additional surgical treatment because of heterotopic ossification that caused a mild limitation of elbow motion in the fixation group, no patient had arthritic changes of the elbow joint in both groups.

## Discussion

The terrible triad injury of the elbow causes extensive damage to bony structures of the elbow and adjacent ligament structures, which result in instability of the elbow joint. Due to its devastating result to elbow stability, surgical treatment is essential to achieve a stable joint for most cases of terrible triad injuries [3, 17]. The treatment goal of such an injury is to restore sufficient elbow joint stability to enable a stable arc of motion [1, 3, 7, 8, 15]. Most studies agreed that fixing or replacing the radial head and repairing the lateral ligamentous complex are necessary to achieve stability [3, 7–9]. However, there are many debates about the necessity to fix the relatively small coronoid fractures in cases of terrible triad injuries. In addition, it would be beneficial for surgeons if coronoid fracture fixation was not needed because this procedure is challenging to perform. Although the controversy over whether to fix coronoid fractures still exists, many authors advocate that the associated coronoid process fracture, regardless of the fragment size, should also be fixed [4, 8, 9, 12, 18, 19]. Otherwise, some recent studies had reported that stability could be restored without coronoid process fixation in Regan-Morrey type I and II fractures, especially those with intact lateral structures [8, 15, 20]. In particular, one study based on cadaveric evaluation reported that stability was maintained when both the radial head was intact and coronoid deficiency was less than 40% [15].

Until now, a clear surgical protocol for terrible triad injuries remains unclear, but it is generally agreed that the motion of a functional elbow should be restored while ensuring sufficient stability with a minimally invasive method. Furthermore, as noted in the results of this study, the clinical outcomes measured by the elbow functional score and ROM were excellent in both the fixation and the non-fixation groups. Therefore, we deduced that coronoid fractures accompanied by terrible triad injuries under several clarified conditions could be managed well without surgical fixation.

In the present study, three patients (two, fixation group; one, non-fixation group) were treated for additional medial structure injuries mainly due to common flexor injury. Although MCL injury is also a common comorbid injury in terrible triad injuries, to date, it is also controversial whether treatment of the MCL should be performed [18, 21, 22]. Based on the present study, we suggest that if common flexor origin rupture is accompanied with MCL injury, surgical treatment for medial structures may be necessary regardless of the size of the coronoid deficiency. However, if the common flexor origin is intact, it is thought that the decision of an additional surgery for MCL injury and coronoid fracture can be based on the amount of coronoid deficiency and the result of the intraoperative elbow stability test after the restoration of the lateral structures.

In radiologic evaluation performed during the follow-up period, heterotopic ossification was observed in one patient in the fixation group. Except this occurrence, no specific findings such as post-traumatic arthrosis were found in either group. However, considering the study design, the mean age of the patients included in this study (54.2 years), and the mean follow-up period (57.1 months), a study with a long-term follow-up period is warranted for precise evaluation of post-traumatic arthrosis.

This study had several limitations. The number of patients in each group was small; we did not compare whether coronoid fixation should be performed in cases with the same degree of coronoid deficiency. The study was performed retrospectively at a single center by one surgeon.

However, we believe that it is a meaningful study in that the comparison of clinical outcomes, such as elbow ROM and functional score, depending on whether coronoid fixation was performed in terrible triad injuries, is helpful in showing that coronoid fixation can be avoided in such cases.

## Conclusions

We observed no significant differences in clinical outcomes between the fixation and non-fixation groups in terrible triad injuries. Therefore, we deduced that if patients with a terrible triad injury with a height loss of less than 50% can maintain elbow stability after the restoration of the radial head and LUCL injuries, internal fixation of the coronoid process fracture may not be necessary.

## Data Availability

The datasets used and/or analyzed during the current study are available from the corresponding author on reasonable request.

## Abbreviations

CT: Computed tomography
LUCL: Lateral ulnar collateral ligament
MCL: Medial collateral ligament
MEPS: Mayo Elbow Performance Score
MRI: Magnetic resonance imaging
ROM: Range of motion
